# Every angle covered: osWRKY36 regulates leaf inclination in rice

**DOI:** 10.1093/plcell/koag129

**Published:** 2026-05-06

**Authors:** Nataliia Konstantinova

**Affiliations:** Assistant Features Editor, The Plant Cell, American Society of Plant Biologists; Center for Plant Systems Biology, VIB, Ghent B-9052, Belgium; Department of Plant Biotechnology and Bioinformatics, Ghent University, Ghent B-9052, Belgium

What is the best angle to catch a bit of sun when sitting at your desk? For monocots, leaf angles are directly linked to photosynthetic efficiency, which in turn affects crop production. The lamina joint, connecting the leaf blade and sheaf, plays a prominent role in determining the leaf angle (Cao and Chen 1995). Brassinosteroid (BR)-governed proliferation and elongation of cells on adaxial and abaxial sides of the lamina joint determines how inclined or erect the leaves are, with enhanced BR signaling leading to more inclined leaves. In addition to BRs, WRKY transcription factors have also been studied in the context of plant growth and development (Wang et al. 2023), but more mechanistic insight is needed to understand their role in leaf blade angle formation.

**Figure 1:**
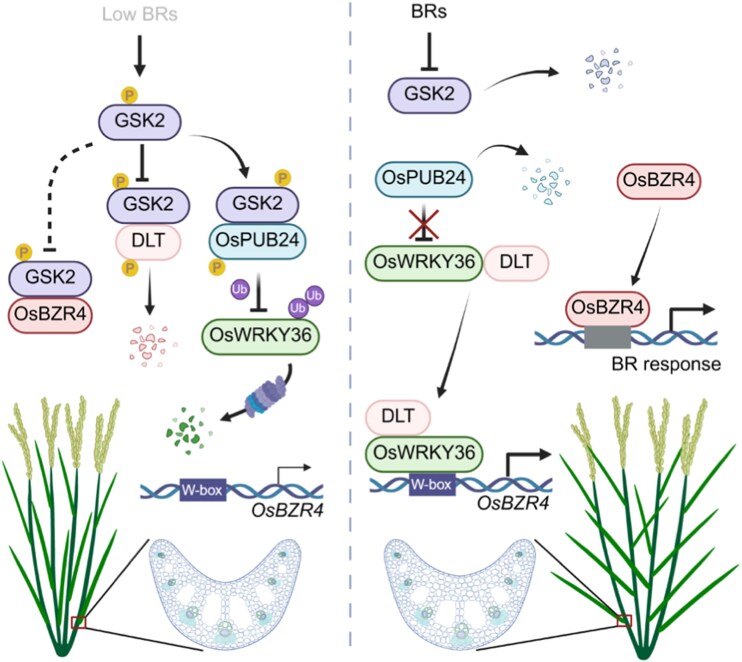
Model summarizing OsWRKY36-mediated leaf inclination via BRs. Left: in absence of BR, GSK2 phosphorylates OsBZR4, DLT, and OsPUB24, which in turn leads to degradation of OsWRKY36 and keeps OsBZR4 at low concentrations. This limits the growth and elongation of adaxial parenchyma cells and restricts leaf inclination. Right: BRs inhibit activity of GSK2 and thus promote leaf inclination by preventing OsWRKY36 degradation (via OsPUB24 inhibition), enabling OsWRKY36–DLT to activate OsBZR4 and enhance BR-responsive growth that leads to increased leaf angles. Adapted from Zhao et al (2026), Figure 7G.

By in-depth characterization of OsWRKY36, Zhao and colleagues (2026) unveil new transcriptional and post-translational mechanistic components controlling leaf angle in rice (Fig. 1).

The authors first conducted a screen using a CRISPR-Cas9–based mutant library targeting *OsWRKY* genes in the rice Nip background. They found that the *Oswrky36* mutant displayed a reduced leaf angle and a more compact overall morphology. To validate that *OsWRKY36* positively regulates rice leaf inclination, CRISPR mutant lines targeting *OsWRKY36* and overexpression (OE) lines were generated and analyzed for leaf angle and growth phenotypes. *Oswrky36* mutants produced smaller leaf angles, while the *OsWRKY36* OE lines had the opposite: increased leaf angles. Furthermore, functional complementation fully rescued the reduced leaf angle phenotype of the mutant, validating the role of *OsWRKY36* in this process.

RT-qPCR and GUS staining showed high expression of *OsWRKY36* in leaf lamina joints, while RNA in situ hybridization confirmed expression specifically in the parenchyma cells and sclerenchyma clusters. When taking a closer look at the lamina joints of wild-type Nip plants, *oswrky36* mutants, and the *OsWRKY36* OE lines, the authors found that the adaxial parenchyma cell layers were shortened, fewer, and thinner in the mutant. Conversely, the OE lines showed elongated, increased, and enlarged adaxial parenchyma cells. This suggests that by regulating the expansion of adaxial parenchyma cells in the lamina joint, OsWRKY36 influences leaf angles. Because BRs are known to play a key role in regulating leaf angles (Cao and Chen 1995), the authors next examined the responses of wild-type, *oswrky36* mutants, and the *OsWRKY36* OE lines to different concentrations of 24-epibrassinolide (the most active form of BRs). Mutants were hyposensitive, while OE showed enhanced sensitivity, suggesting that OsWRKY36 positively regulates BR-induced leaf inclination.

To understand the role of OsWRKY36 in BR signaling, the authors performed a yeast 2-hybrid screen to identify its putative interactors. Among the candidates, they focused on those that encoded BR signaling components: OsPUB24 and DLT. These interactions were validated using co-IP and BiFC assays, while RNA in situ hybridization revealed that OsPUB24 and DLT are expressed in the rice lamina joint, indicating a potential spatial interaction. Because OsPUB24 is an E3 ubiquitin ligase, the authors performed a series of biochemical assays demonstrating that this protein ubiquitinates OsWRKY36, targeting it for degradation via the 26S proteasome. Both OsPUB24 and DLT previously were shown to interact with GSK2, a negative regulator of BR signaling (Min et al. 2019). Although the authors found no direct interaction of GSK2 with OsWRKY36, these components could nevertheless be part of the same regulatory network regulating BR signaling.

Finally, to identify direct transcriptional targets of OsWRKY36, the authors performed complementary RNA, DAP-seq, and ChIP-seq analysis using the *Oswrky36* mutant and the OsWRKY36 OE lines. This revealed OsBZR4, previously shown to play a role in regulating BR signaling and rice leaf inclinations, as a potential target (Liu et al. 2021). To support their model, the authors showed that OsWRKY36 directly binds the *OsBZR4* promoter, with coexpression of DLT increasing OsWRKY36-mediated *OsBZR4* promoter activity, while OsPUB24 suppressed it. Consistent with this, *osbzr3osbzr4* double mutant and the OE lines showed decreased BR sensitivity and increased BR sensitivity, respectively.

In summary, the authors present an elegant model regulating rice leaf inclinations. They show that BRs inhibit OsPUB24 from degrading OsWRKY36, which allows OsWRKY36 to interact with DLT and drive *OsBZR4* expression, positively regulating leaf inclination.

**Recent related articles in *The Plant Cell*:**

- Liu et al. (2024) described how the rice transcription factor FOUR LIPS expressed in the lamina joint connects brassinosteroid signaling, lignin deposition, and leaf angle.
- Yue et al. (2024) showed how 2 variants of WIDTH OF LEAF AND GRAIN differ in their ubiquitination activity of LARGE2, which in turn affects rice grain and leaf size.
- Sheng et al. (2025) characterized a maize GRAS protein that interacts with an AP2 transcription factor to control leaf angle and plant height.
